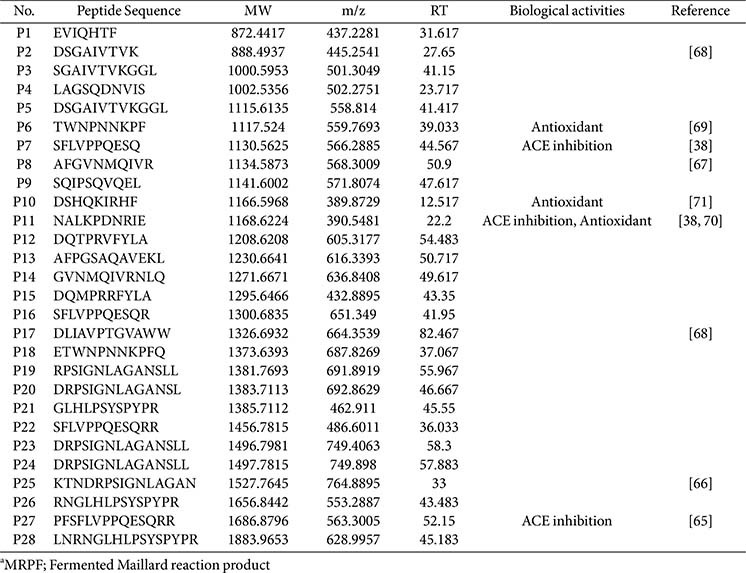# Fermented Soy Protein Maillard Product Prevents Bone Loss via TNF-α Suppression and Gut–Bone Axis Modulation in Ovariectomized Mice

**DOI:** 10.4014/jmb.2511.11004

**Published:** 2025-12-29

**Authors:** Hyun Jin Bae, Jae Yeon Joung, Hyo Su Choi, Ji Seung Han, Jin Hwan Kim, Jae Kyeom Kim, Nam Su Oh

**Affiliations:** 1 Department of Food and Biotechnology, Korea University, Sejong 30019, Republic of Korea; 2 College of Life Sciences and Biotechnology, Korea University, Seoul 02841, Republic of Korea; 3 Institute of Life Sciences and Natural Resources, Korea University, Seoul 02841, Republic of Korea; 4 Department of Health Behavior and Nutrition Sciences, University of Delaware, Newark, DE 19701, USA

**Keywords:** Bioactive peptide, gut microbiota, inflammation, *Lacticaseibacillus rhamnosus* IM18, bone resorption, maillard reaction

## Abstract

This study investigated the bone-protective effects of Maillard reaction products (MRPs) from isolated soy protein and their fermentation product (MRPF) using *Lacticaseibacillus rhamnosus* IM18. In lipopolysaccharide-induced RAW264.7 cells, MRP showed enhanced antioxidant and anti-inflammatory activities compared to isolated soy protein, which were further improved by MRPF. In ovariectomized mice and RANKL-stimulated RAW264.7 cells, MRPF demonstrated superior anti-osteoclastogenic and bone-protective effects by suppressing osteoclast differentiation and preventing bone resorption. These effects involved downregulation of pro-inflammatory cytokines (*Tnfa*, *Il1b*, *Il6*, and *Tnfs11*) and upregulation of osteoprotegerin (Tnfrsf11b), along with restoration of intestinal barrier genes (*Ocln*, *Cldn1*, and *Tjp1*). MRPF administration significantly modulated gut microbiota, reducing inflammation-associated taxa (*Desulfovibrio*) while enriching beneficial genera (*Bifidobacterium*, *Ruminococcus*, and *Akkermansia*). Peptide profiling identified 28 bioactive peptides contributing to observed effects. These findings indicate that MRPF alleviates inflammation and maintains gut homeostasis, supporting its potential as a functional food ingredient for postmenopausal osteoporosis.

## Introduction

Osteoporosis is a bone metabolic disorder characterized by an imbalance in bone remodeling, leading to decreased bone mineral density (BMD) and disruption of skeletal microstructure, thereby increasing the risk of fragility fractures [1]. Osteoporosis is commonly classified into postmenopausal osteoporosis, predominantly associated with estrogen deficiency, and senile osteoporosis, which arises due to physiological aging processes [2, 3]. Among these, estrogen deficiency following menopause is a well-established risk factor for osteoporosis, because of its capacity to disrupt bone remodeling balance. Estrogen plays a crucial role in maintaining bone homeostasis by suppressing osteoclast differentiation and modulating the production of inflammatory mediators. Estrogen deficiency leads to the upregulation of pro-inflammatory cytokines, including tumor necrosis factor-alpha (TNF-α), interleukin-1 beta (IL-1β), and interleukin-6 (IL-6), which promote osteoclastogenesis via activation of the receptor activator of nuclear factor-κB ligand (RANKL) signaling pathway [4-6]. The upregulation of these cytokines induces a state of chronic low-grade inflammation, often referred to as osteoimmunological dysregulation, which accelerates bone resorption and contributes to progressive loss of bone mass in postmenopausal women [7, 8].

Among the pro-inflammatory cytokines elevated under estrogen-deficient conditions, TNF-α is pivotal in promoting osteoclastogenesis by enhancing RANKL expression and stimulating osteoclast precursor differentiation [9]. Beyond its bone- resorptive effects, TNF-α also mediates systemic inflammation and has been implicated in the pathogenesis of various chronic diseases, including rheumatoid arthritis, inflammatory bowel disease, metabolic syndrome, and cardiovascular disease [10-12]. Therefore, targeting TNF-α signaling could be a promising therapeutic approach for alleviating estrogen deficiency-induced bone loss and inflammation-associated comorbidities in postmenopausal populations [13].

Isolated soy protein (ISP) is a high-quality plant protein that contains essential amino acids, including lysine, leucine, and valine, making it a valuable alternative to animal-based proteins for maintaining a balanced diet [14]. The two major proteins in soy, glycinin (11S globulin) and β-conglycinin (7S globulin), together account for more than 70% of total soy protein and have been reported to exhibit a wide range of bioactivities, including antioxidant, hypocholesterolemia, and anti-inflammatory effects [15]. In particular, glycinin-derived peptides have demonstrated strong radical-scavenging capacity, indicating superior antioxidant potential [16]. In addition, β-conglycinin peptides have shown anti-inflammatory properties by suppressing inducible nitric oxide synthase (iNOS)/nitric oxide and cyclooxygenase-2/prostaglandin E_2_ signaling pathways in lipopolysaccharide (LPS)-stimulated macrophages [17]. However, the application of ISP is limited by its structural constraints and variability in functionality. Consequently, several modification techniques, including enzymatic hydrolysis, ultrasound treatment, microbial bioconversion, and the Maillard reaction, have been explored to enhance the physicochemical and functional properties of plant proteins [18].

The Maillard reaction is a non-enzymatic glycosylation reaction between amino groups in proteins and carbonyl groups in reducing sugars. It is widely used in food processing as a safe and promising method for protein modification, eliminating the need for chemical additives. Protein–sugar conjugates formed through the Maillard reaction enhance the functional properties of dietary proteins by reducing allergenicity, improving antioxidant and anti-inflammatory activities, and improving physicochemical properties such as emulsifying, gelling, and stabilizing abilities [19, 20]. Gu *et al*. [21] attributed the enhanced reducing capacity of Maillard reaction products (MRPs) to specific chemical structures formed during the reaction, including heterocyclic compounds and hydroxyl groups. Furthermore, our previous study showed that fermenting casein-derived MRPs with *L. rhamnosus* yielded bioactive peptides with enhanced antioxidant, anti-thrombotic, and anti-inflammatory activities, which also ameliorated cognitive impairment in chronic mild stress-induced mice [22-24]. However, despite these promising bioactivities, the potential of fermented soy protein MRPF to mitigate bone resorption in the context of postmenopausal osteoporosis remains unexplored.

To this end, in this study, we aimed to investigate the protective effects of a dietary fermented MRP (MRPF) derived from soy protein on bone health in an ovariectomy (OVX)-induced mouse model of postmenopausal osteoporosis. Specifically, we evaluated whether MRPF supplementation alleviates bone resorption by suppressing TNF-α and modulating gut microbiota composition. This study may offer insights into the potential use of MRPF as a functional food ingredient for preventing inflammation-associated disorders.

## Materials and Methods

### Materials

Glucose was purchased from the Junsei Chemical Co. (Japan). Sodium dodecyl sulphate (SDS) and 2,2-diphenyl-1-picrylhydrazyl (DPPH) were obtained from Thermo Fisher Scientific Inc. (USA). All other chemicals and solvents were purchased from Sigma–Aldrich Co., Ltd. (USA).

### Preparation of Samples

**Preparation of MRP.** A commercial ISP (PRO-FAM 974, containing a minimum of 90% protein, maximum of 6% moisture, 4% fat, and 5% ash) was obtained from Archer Daniels Midland Co. (USA). The ISP comprised essential amino acids, including leucine (8 g 100 g^−1^ protein), lysine (6.4 g 100 g^−1^ protein), phenylalanine (5.4 g 100 g^−1^ protein), valine and isoleucine (4.8 g 100 g^−1^ protein), as well as threonine, histidine, and tryptophan (as reported by the manufacturer). The ISP (5%, w/v) was dissolved in distilled water, and the pH was adjusted to 10 using 1 mol L^−1^ NaOH. Subsequently, the solution was heated in a water bath at 90°C for 20 min without agitation. After cooling in a cold-water bath, glucose (5%, w/v) was added, and the pH was adjusted to 6.9 using 1 mol L^−1^ HCl while stirring the mixture at room temperature using a magnetic stirrer. Subsequently, the Maillard reaction was carried out at 70°C in a water bath for 18 h with continuous shaking.

**Preparation of MRPF.** In our previous investigation approved by the Institutional Review Board of Korea University (KUIRB-2021-0116-02), novel lactic acid bacteria (LAB) strains were isolated from the feces of healthy infants under two weeks of age. Eight strains including two strains of *Lactobacillus gasseri*, one strain of *Limosilactobacillus fermentum*, three strains of *Lacticaseibacillus rhamnosus*, and two strains of *Limosilactobacillus reuteri* were evaluated for their fermentation with MRP. Of these, *L. rhamnosus* IM18, which showed the highest antioxidant activity (Data not shown), was selected in this study. The strain was activated three times in de Man–Rogosa –Sharpe broth (Difco, USA) at 37°C for 18 h each, followed by two washes and resuspension in 10 mM sodium phosphate buffer (pH 7.2). The MRP solution was pasteurized in a hot-water bath at 80°C for 10 min and immediately cooled in cold water. The cooled solution was then inoculated with 5 ml L^−1^ of *L. rhamnosus* IM18 and incubated at 37°C for 48 h. All samples were stored at−80°C for subsequent analysis.

### Sodium Dodecyl Sulfate-Polyacrylamide Gel Electrophoresis (SDS-PAGE)

SDS-PAGE was performed using 12% and 4% acrylamide separating and stacking gels, respectively [25]. Each sample was mixed with 2× Laemmli sample buffer (Bio-Rad, USA) at a 1:1 ratio and denatured at 100°C for 5 min. Electrophoresis was conducted at 100 V for 2 h, and the gel was stained for 1 h with Coomassie Brilliant Blue R-250 (Bio-Rad).

### Degree of Hydrolysis

The free-amino-acid content was evaluated using the ortho-phthalaldehyde (OPA) assay based on a previously reported method [26], with slight modifications. Samples were centrifuged (10,000 ×*g*, 10 min, 4°C), and 10 μl of the supernatant was mixed with 180 μl of the OPA reagent. After incubation for 2 min at room temperature the absorbance was measured at 340 nm using a microplate reader (BioTek Instruments, USA). A standard curve was constructed using L-lysine (Thermo Fisher Scientific, USA).

### Peptide Identification Using Liquid Chromatography–Tandem Mass Spectrometry (LC-MS/MS) Analysis

Peptides were extracted from MRPF following lipid removal using a modified Folch method [27]. The aqueous phase containing peptides was collected, and proteins were precipitated using 12% trichloroacetic acid. The supernatant was desalted and neutralized using a Sep-Pak C18 cartridge (Waters Co., USA), filtered through a 3 kDa molecular weight cut-off membrane, and lyophilized for analysis.

LC-MS/MS analysis was conducted using a Dionex Ultimate 3000 UHPLC system (Thermo Fisher Scientific) coupled with a TripleTOF 5600 mass spectrometer (AB SCIEX, USA). Peptides were separated on an ACQUITY UPLC BEH C18 column (130 Å, 1.7 μm, 2.1 × 100 mm; Waters Co.) using a linear gradient of 1–100% acetonitrile containing 0.1% formic acid over 106 min at 300 μl min^−1^. A 2 μl sample volume was injected in split mode (30:1). MS acquisition was performed in positive ion mode with the following parameters: curtain gas, 25 psi; ion source gases 1 and 2, 50 psi each; ion spray voltage floating, 5,500 V; and source temperature, 500°C. Mass spectra were acquired in the m/z range of 300–2,500, with a collision energy of 44 V and declustering potential of 80 V. Peptides were identified using ProteinPilot and PeakView software (AB SCIEX), and MS/MS spectra were matched to a digested peptide library constructed from Glycine max protein sequences. High sequence coverage was obtained for major soybean storage proteins, including glycinin G2 (P04405), glycinin G4 (P02855), and β-conglycinin α-subunit (Q4LER5), confirming that the peptides originated from proteolytic cleavage of soy proteins by *L. rhamnosus* during MRPF fermentation.

### Antioxidant Activity

A ferric-reducing antioxidant power (FRAP) assay was performed to verify the reducing abilities of ISP, MRP, and MRPF. Radical scavenging activities were assessed using DPPH, 2, 2'-azino-bis-(3-ethylbenzothiazoline-6-sulfonate) (ABTS), and hydroxyl (OH) radical scavenging assay, following protocols described in previous studies [22, 28].

### Anti-Inflammatory Activity

Murine macrophage cell line RAW 264.7 was obtained from the Korean Cell Line Bank (Republic of Korea) and maintained in Dulbecco’s modified Eagle’s medium (Gibco, Thermo Fisher Scientific, USA) supplemented with 10% fetal bovine serum (FBS; Gibco) and 1% penicillin–streptomycin (Gibco) at 37°C in a humidified atmosphere containing 5% CO_2_. Cells were seeded at a density of 1.5 × 10^4^ cells per well in 96-well plates and incubated for 18 h. Subsequently, samples were added at 10 μg ml^−1^ and incubated for 12 h before stimulation with LPS (100 ng ml^−1^) for an additional 12 h.

### Quantitative Real-Time PCR (qRT-PCR) Analysis

The total RNA was extracted from cells and tissues of mice using the TRIzol reagent (Invitrogen, USA), according to the manufacturer’s instructions. cDNA was synthesized from 1 μg of total RNA using BioFACT 2X Reverse Transcription Pre-Mix (BioFACT, Republic of Korea). qRT-PCR was performed with GoTaq qPCR Master Mix (Promega Co, USA) using a QuantStudio 3 Real-Time PCR system (Applied Biosystems, USA). The level of relative mRNA expression was calculated using the 2^−ΔΔCT^ method [29] and normalized to the housekeeping gene (*Gapdh*). The primer sequences used for amplification are listed in Table S1.

### Osteoclast Differentiation and Tartrate-Resistant Acid Phosphatase (TRAP) Staining

RAW 264.7 cells were seeded at a density of 2 × 10^4^ cells per well in 24-well plates and incubated for 24 h to induce osteoclast differentiation. The medium was then replaced with alpha-MEM (HyClone, USA) supplemented with 10% FBS, 1% penicillin–streptomycin, and 50 ng ml^−1^ of RANKL (PeproTech, USA). For treatment groups, samples were added to the differentiation medium at a final concentration of 10 μg ml^−1^, and the medium was refreshed every two days. On day 5, osteoclast differentiation was evaluated using the TRACP and ALP double-stain kit and TRAP & ALP activity kit (Takara, Japan), according to the manufacturer’s instructions. Representative images were acquired using a light microscope (EVOS XL Core, Invitrogen, UK). Quantification of TRAP-positive areas was performed using a color-based pixel thresholding method implemented in Python (OpenCV), utilizing hue-saturation-value (HSV) color space masking. Pixels within the defined hue range for reddish to purplish staining (H: 120–180; S: 50–255; V: 50–255) were classified as TRAP-positive. The total cell-covered area was estimated by applying grayscale thresholding. The percentage of TRAP-positive areas relative to the total cell area was calculated for each image.

### Ovariectomy-Induced Menopausal Steoporosis Mice

**Mice treatment and sample administration.** C57BL/6N female mice (6 weeks old, 16 ± 1 g) were purchased from Samtako Bio Korea (Republic of Korea) and housed at 22 ± 2°C under a controlled 12-h light/dark cycle with 60 ± 5% humidity. Mice were provided with a standard rodent diet and water *ad libitum*. This study was approved by the Institutional Animal Care and Use Committee of Korea University (IACUC NO. KUIACUC-2021-0015) and all procedures involving animal subjects were conducted in accordance with the guidelines of the Care and Use of Laboratory Animals. After a 2-week acclimation, mice were randomly assigned to four groups (*n* = 6 per group): sham-operated (SHAM), ovariectomized (OVX), ovariectomized and MRP-treated (MRP), and ovariectomized and MRPF-treated (MRPF) groups. Ovariectomy was performed at 8 weeks of age under isoflurane anesthesia, and the SHAM group underwent a sham operation. After a 1-week recovery period, cell-free supernatants of MRP and MRPF (1,500 mg kg^-1^) were administered orally once a day for 14 weeks. A single dose of MRP or MRPF (1,500 mg kg^-1^) was used based on the average food intake reported for this mouse strain and our previous study [22, 30]. The SHAM and OVX groups received equal volumes of phosphate-buffered saline (PBS).

**Micro-computed tomography (micro-CT) analysis.** Microstructural analysis of the distal femur was performed using a Quantum FX micro-CT system (PerkinElmer, USA) to evaluate trabecular bone parameters. Scanning was performed at 90 kVp, 160 μA tube current, and a 10 mm field of view (FOV), with a total scan duration of 3 min. Raw image data were reconstructed and analyzed using Analyze software (PerkinElmer). Sagittal, coronal, and axial plane images were used to identify the endpoint of the distal femoral growth plate. From this reference point, a sub-region of 120 slices (2.4 mm) was defined proximally, following an offset of 30 slices (0.6 mm) to exclude the growth plate. The region of interest was manually selected to include only trabecular bone within the metaphyseal area. A mask map was generated to isolate trabecular structures, and the following parameters were calculated: BMD (g/cm^3^), trabecular number (Tb.N; 1/mm), connectivity density (Conn.D; 1/mm^3^), trabecular separation (Tb.Sp; mm), and bone volume/total volume (BV/TV; %).

**Histological analysis.** Colon tissues were collected, rinsed with cold PBS, and fixed in 4% paraformaldehyde (Biosesang; Seongnam, Republic of Korea) for 24 h at 4°C. The fixed tissues were embedded in paraffin, and 5 μm-thick sections were prepared using a Leica RM2155 rotary microtome (Leica Microsystems; Nussloch, Germany). For histological evaluation, tissue sections were stained with hematoxylin and eosin (H&E) following standard protocols [31].

**Fecal microbiota analysis.** Fecal samples were collected from each mouse and immediately stored at −80°C until further processing. Genomic DNA was extracted using a QIAamp DNA Stool Mini Kit (Qiagen, Germany), following the manufacturer’s protocol. The V3–V4 hypervariable regions of the 16S rRNA gene were amplified and sequenced on an Illumina MiSeq platform. Raw sequencing reads were processed using the QIIME2 pipeline (version 2024.10). Demultiplexed sequences were trimmed, denoised, and merged using DADA2 to obtain high-quality feature tables. Low-quality reads were filtered, and the sequences were trimmed to 280 bases for forward reads and 220 bases for reverse reads before merging. Amplicon sequence variants (ASVs) were taxonomically assigned using a Naïve Bayes classifier trained on the SILVA 138.2 reference database (silva-138-2-99-nb-classifier.qza). Microbial composition was visualized at the phylum, family, and genus levels. Beta diversity was assessed using principal coordinates analysis (PCoA) based on weighted UniFrac distance matrices. Differentially abundant taxa across groups were identified using linear discriminant analysis effect size (LEfSe), and results are presented as an Linear Discriminant Analysis (LDA) score histogram.

**Short-chain fatty acids (SCFAs) analysis.** Approximately 20 mg of fecal sample was mixed with 50 μl of 50% (v/v) sulfuric acid, vortexed for 3 min, and, 11 μl of heptanoic acid (internal standard) was added. Subsequently, 200 μl ethyl ether was added to the mixture, followed by centrifugation at 2,800 ×*g* for 5 min. The resulting upper organic layer containing SCFAs was carefully transferred into a fresh microtube. This extraction procedure was repeated three times, and the pooled ether fractions (600 μl total) were used for SCFA analysis. SCFAs were quantified and identified following the method of Scortichini *et al*. [32], with minor modifications. Seven SCFAs—including acetic acid, propionic acid, caproic acid, isobutyric acid, butyric acid, isovaleric acid, and valeric acid—were analyzed using a gas chromatograph (7890A GC, Agilent Technologies, USA) equipped with a flame ionization detector (FID). Separation was performed using a DB-FFAP capillary column (30 m × 0.32 mm, 0.25 μm film thickness; Agilent). Samples were injected in split mode (30:1 ratio), with an injection volume of 2 μl. The GC oven program was as follows: initial temperature at 40°C for 2 min, increased at a rate of 12°C min^−1^ to 65°C, then further ramped at 10°C min^−1^ to 245 °C, with a total run time of 21.5 min. The FID was maintained at 250°C, and helium was used as the carrier gas at a constant flow rate of 1.20 ml min^−1^.

### Statistical Analysis

For statistical analysis, data are presented as mean ± standard deviations. We first assessed if the data of each endpoint followed a normal distribution using the Shapiro-Wilk test. Comparisons between two groups were assessed using the Student’s *t*-test while multiple groups were compared via one-way analysis of variance. The statistical analyses were performed using the IBM SPSS Statistics (version 26.0, IBM, USA) and a *p*-value of < 0.05 were considered statistically significant.

## Results

### Protein Profiling and Peptide Characterization

**Fig. 1:**
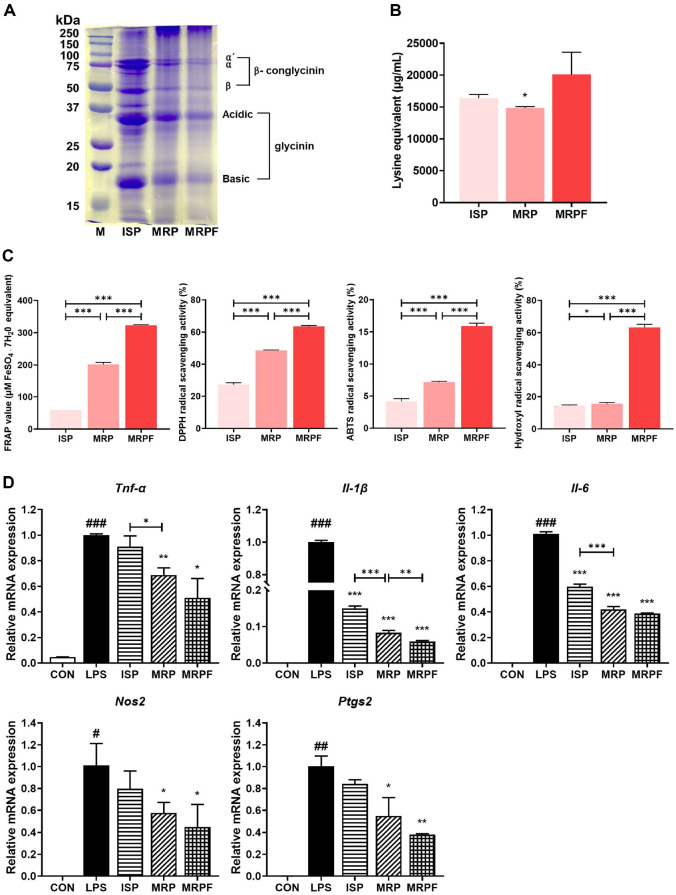
Protein profile, antioxidant, and anti-inflammatory effects of ISP, MRP and MRPF. (**A**) SDS-PAGE patterns of ISP, MRP, and MRPF. (**B**) Degree of hydrolysis measured using OPA assay with L-lysine as a standard. (**C**) Antioxidant activities evaluated using FRAP, and DPPH, ABTS, and hydroxyl radical scavenging assays. FRAP values were calculated using the FeSO_4_ standard curve. (**D**) Anti-inflammatory effects assessed in LPS-stimulated RAW 264.7 macrophages by measuring the mRNA expression of *Tnfa*, *Il1b*, *Il6*, *Nos2*, and *Ptgs2* using qRT-PCR. Data are presented as mean ± standard deviation (n = 3). *p < 0.05, **p < 0.01, ***p < 0.001; ^#^*p* < 0.05, ^##^*p* < 0.01, ^###^*p* < 0.001 vs. control. Statistical differences between two groups were analyzed using Student’s t-test, and multiple group comparisons were performed using one-way analysis of variance. M, molecular weight marker; ISP, isolated soy protein; MRP, soy protein Maillard reaction product; MRPF, fermented Maillard reaction product; OPA, ortho-phthalaldehyde; FRAP, ferric reducing antioxidant power; DPPH, 2,2-diphenyl-1-picrylhydrazyl; ABTS, 2,2'-azino-bis(3-ethylbenzothiazoline-6-sulfonic acid).

SDS-PAGE analysis revealed distinct changes in the protein profiles following Maillard reaction and subsequent fermentation (Fig. 1A). In the MRP group, bands corresponding to glycinin and β-conglycinin appeared thinner than that in the ISP group. Nevertheless, a pronounced high-molecular-weight band above 250 kDa was observed in the MRP group, indicating the formation of macromolecular aggregates via the Maillard reaction. In contrast, MRPF group exhibited overall weaker band intensities than MRP group, indicating hydrolysis of the proteins by *L. rhamnosus* IM18 and its secreted proteases during fermentation.

Next, we quantified the degree of hydrolysis using the OPA method to assess the extent of protein hydrolysis (Fig. 1B). The MRP group showed slightly reduced lysine content compared to ISP, indicating lysine involvement in the Maillard reaction. However, MRPF exhibited a significantly higher amount of free amino acids than ISP and MRP, confirming extensive protein degradation during fermentation.

A total of 28 peptides were identified from MRPF using LC-MS/MS analysis, as visualized in the overlaid extracted ion chromatogram (Fig. S1). These peptides were detected across a broad retention time range (12.5–82.5 min) with molecular weights ranging from 872 to 1,884 Da and m/z values from 389 to 750. Each peak was annotated based on retention time, and peptide sequences are listed in Table 1. Fifteen of these 28 peptides were derived from glycinin, particularly from glycinin G2 and G4 subunits, while others originated from the β-conglycinin α-subunit and minor soy protein components.

### Antioxidant and Anti-Inflammatory Activities of ISP, MRP, and MRPF

As shown in Fig. 1C, MRP showed significantly higher reducing power (208.11 μmol FeSO_4_·7H_2_O equivalents) than ISP (59.78 μmol FeSO_4_·7H_2_O equivalents) (*p* < 0.001), while MRPF exhibited the highest FRAP values (326.44 μmol FeSO_4_·7H_2_O equivalents). Similarly, the DPPH and ABTS radical-scavenging capacities increased significantly after the Maillard reaction and were further enhanced after fermentation (*p* < 0.001). Furthermore, the hydroxyl radical scavenging activity increased to 63.20% in MRPF, compared to 15.67% in MRP (*p* < 0.001).

The anti-inflammatory potential of MRP and MRPF was evaluated in LPS-stimulated RAW 264.7 macrophages (Fig. 1D). LPS treatment significantly increased the expression of pro-inflammatory genes compared to the control treatment. qRT-PCR analysis revealed that treatment with the MRP significantly downregulated the relative mRNA levels of *Tnfa*, *Il1b*, *Il6*, *Nos2*, and *Ptgs2*. The expression of these genes was further reduced in the MRPF-treated sample, indicating that fermentation enhanced the anti-inflammatory properties.

### Inhibitory Effects of MRP and MPRF on Osteoclast Differentiation in RANKL-Induced RAW264.7 Macrophage

**Fig. 2:**
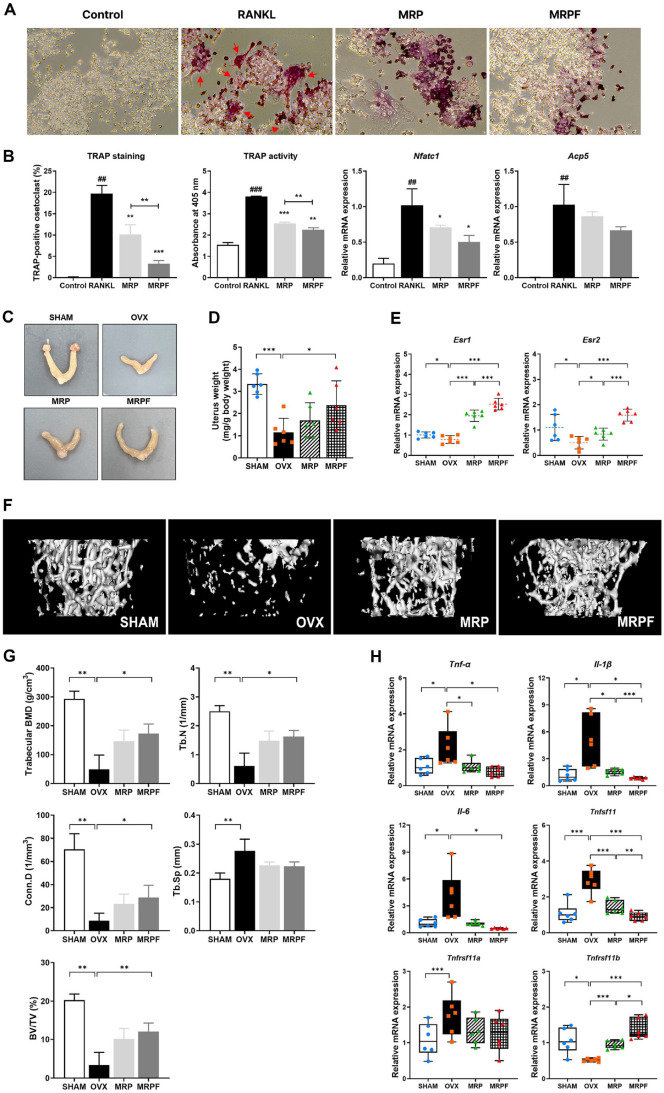
*In vitro* anti-osteoclastogenic effects and *in vivo* estrogenic and protective effect on OVX-induced bone loss in mice. (**A**) Representative TRAP-stained images of RAW 264.7 cells treated with RANKL alone or in combination with MRP or MRPF (red arrows: TRAP-positive multinucleated cells). (**B**) Quantification of TRAP-positive cells, TRAP activity, and mRNA expression levels of osteoclast differentiation markers (*Nfatc1* and *Acp5*) to evaluate the inhibitory effects of MRP and MRPF on RANKL-induced osteoclastogenesis. (**C**) Representative images of uterine tissues to assess uterine atrophy and evaluate the protective effects of MRP and MRPF in OVX mice. (**D**) Uterine weights measured to confirm the efficacy of OVX surgery and potential estrogen-like effects of MRP and MRPF. (**E**) mRNA expression levels of estrogen receptors (*Esr1* and *Esr2*) in uterine tissue analysed using qRT-PCR. (**F**) Representative micro-CT images of distal femur trabecular regions from each group. (**G**) Trabecular bone parameters—including bone mineral density (BMD; g/cm^3^), trabecular number (Tb.N; 1/mm), connectivity density (Conn.D; 1/mm^3^), trabecular separation (Tb.Sp; mm), and bone volume / total volume (BV/TV; %)— quantified to evaluate bone structural integrity. (**G**) Expression of bone inflammationrelated (*Tnf-α*, *Il-1b*, and *Il-6*) and bone resorption-related (*Tnfsf11*, *Tnfrsf11a*, and *Tnfrsf11b*) genes in the distal femur measured using qRT-PCR. Data are presented as mean ± standard deviation (n = 3). *p < 0.05, **p < 0.01, ***p < 0.001; ^#^*p* < 0.05, ^##^*p* < 0.01, ^###^*p* < 0.001 vs. control. Statistical differences between two groups were analyzed using Student’s t-test, and multiple group comparisons were performed using one-way analysis of variance. OVX, ovariectomized; TRAP, tartrate-resistant acid phosphatase; RANKL, receptor activator of nuclear factor-κB ligand; MRP, soy protein Maillard reaction product; MRPF, fermented Maillard reaction product.

The anti-osteoclastogenic effects of MRP and MRPF were investigated in RANKL-stimulated RAW 264.7 cells using TRAP staining, TRAP activity assays, and gene expression analysis (Fig. 2A and 2B). RANKL stimulation induced osteoclast differentiation, as evidenced by a significant increase in TRAP-positive multinucleated cells, elevated TRAP activity, and upregulation of osteoclast-related markers including nuclear factor of activated T cells 1 (*Nfatc1*), and acid phosphatase 5 (*Acp5*), compared with the control treatment (*p* < 0.001 and *p* < 0.01, respectively). Both MRP and MRPF treatments suppressed RANKL-induced osteoclastogenesis, with reductions in TRAP-positive cells, TRAP activity, and expression of osteoclast-related genes. MRPF exhibited a greater inhibitory effect than MRP on TRAP staining and TRAP activity.

### Estrogenic Restoration and Bone-Protective Effects of MRP and MRPF in OVX Mice

To evaluate the estrogenic effects, uterine morphology, weight, and estrogen receptor gene expression were assessed in OVX-induced mice. Uterine weight was reduced significantly in the OVX group compared with that in the SHAM group (*p* < 0.001), whereas MRPF supplementation significantly restored uterine weight (*p* < 0.05)(Fig. 2C and 2D). Furthermore, qRT-PCR analysis revealed that the expression levels of *Esr1* and *Esr2* were significantly downregulated in the OVX mice compared with those in the SHAM group (*p* < 0.05; Fig. 2E). In contrast, the expression of these estrogen receptor genes increased significantly in the MRP (*p* < 0.05) and MRPF (*p* < 0.001) groups compared with those in the OVX group, with the MRPF group showing greater enhancement.

To determine the inhibitory effects on bone resorption of MRP and MRPF, microstructural changes and expression of bone inflammation and resorption-related genes in the femur were analyzed in OVX-induced mice. Micro-CT analysis of femoral trabecular bone (Fig. 2F) revealed substantial bone loss in OVX mice, as evidenced by significant reductions in BMD, Tb.N, Conn.D, BV/TV, and increased Tb.Sp, compared with those in the SHAM group (Fig. 2G; *p* < 0.01 or *p* < 0.001). However, these bone parameters were significantly improved in MRPF-treated mice compared with those in OVX mice (*p* < 0.05 or *p* < 0.01), suggesting enhanced protection against OVX-induced trabecular bone deterioration. As shown in Fig. 2H, OVX surgery markedly increased the expression of inflammation and bone resorption-related genes, including *Tnf-a*, *Il-1b*, *Il-6*, *Tnfsf11* (encodes RANKL), and *Tnfrsf11a* (encodes RANK), while decreasing the expression of *Tnfrsf11b* (encodes OPG), compared with SHAM operation (*p* < 0.05 or *p* < 0.001). Treatment with both MRP and MRPF significantly downregulated *Tnf-a*, *Il-1b*, and *Tnfsf11*, and upregulated *Tnfrsf11b*. MRPF showed higher regulatory effects than MRP on the expression of *Il-1b*, *Tnfsf11*, and *Tnfrsf11b* (*p* < 0.05 or *p* < 0.001), and a significant reduction in *Il-6* expression was observed exclusively in the MRPF-treated group (*p* < 0.05).

### Amelioration of Intestinal Inflammation and Barrier Dysfunction in OVX Mice by MRP and MRPF Treatment

**Fig. 3:**
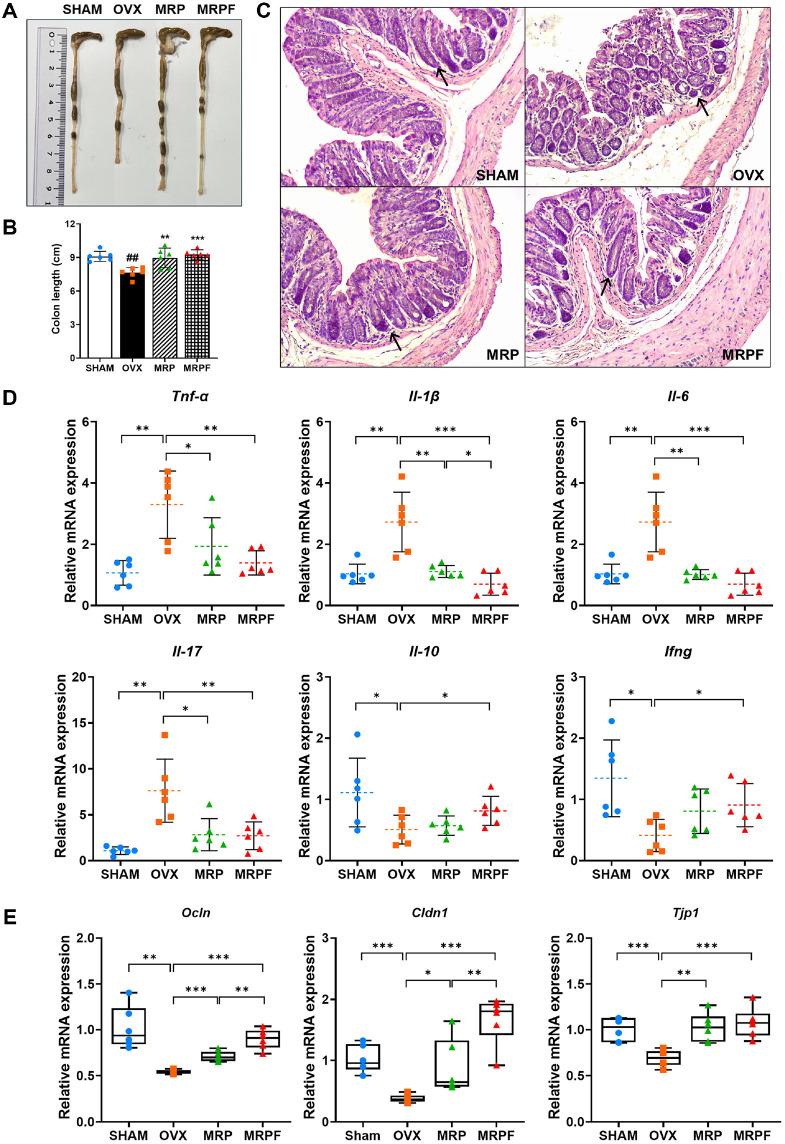
Effects of soy protein Maillard reaction product (MRP) and MRPF on intestinal inflammation, and barrier function in OVX-induced mice. (**A**) Gross morphology of the colon observed to assess phenotypic changes in response to OVX and treatment. (**B**) Tissue atrophy induced by OVX assessed by measuring the colon length. (**C**) Histological analysis of the colon using H&E staining to assess epithelial integrity and inflammatory cell infiltration (black arrows: crypt). The histological analysis was measured using Leica DM500 microscope. (**D**) mRNA expression levels of inflammatory cytokines (*Tnf-a, Il-1b, Il-6, Il-17, Il-10*, and *Ifng*) in the ileum measured using qRT-PCR to assess local inflammation. (**E**) mRNA expression levels of tight junction markers (*Ocln*, *Cldn1*, and *Tjp1*) in the ileum measured using qRT-PCR to evaluate intestinal barrier integrity. Data are presented as mean ± standard deviation (n = 6). *p < 0.05, **p < 0.01, ***p < 0.001; ^#^*p* < 0.05, ^##^*p* < 0.01, ^###^*p* < 0.001 vs. control. Statistical differences between two groups were analyzed using Student’s *t*-test, and multiple group comparisons were performed using one-way analysis of variance. MRP, soy protein Maillard reaction product; MRPF, fermented Maillard reaction product; OVX, ovariectomized; H&E, haematoxylin and eosin.

As shown in Fig. 3A-3B, colon length was significantly reduced in OVX mice compared to that in SHAM mice (*p* < 0.01), indicating colonic atrophy. However, both MRP and MRPF significantly restored colon length (*p* <0.01 and *p* < 0.001). Histological analysis of colon tissue revealed disrupted epithelial thickness and crypts in the OVX group (Fig. 3C). These pathological alterations were notably attenuated by MRP and MRPF supplementation, with improvements in mucosal structure and goblet cell preservation observed in both groups.

Gene expression analysis in the ileum showed that OVX significantly induced intestinal inflammation, as evidenced by increased expression of pro-inflammatory cytokines (*Tnf-a*, *Il-1b*, *Il-6*, and *Il-17*) and reduced expression of the anti-inflammatory cytokines (Il-10 and Ifng) (Fig. 3D). Both MRP and MRPF mitigated these inflammatory changes by suppressing the expression of *Tnf-a*, *Il-1b*, *Il-6*, and *Il-17*. In particular, MRPF showed greater suppression of pro-inflammatory cytokines than MRP, while significantly enhancing *Il10* and *Ifng* expression (*p* < 0.05). OVX also significantly decreased tight junction-related genes, including *Ocln*, *Cldn1*, and *Tjp1* (*p* < 0.01 or *p* < 0.001 vs. SHAM; Fig. 3E). However, treatment with MRP and MRPF significantly upregulated these genes, with MRPF showing a slightly greater restoration of *Ocln* and *Cldn1* levels than MRP (*p* < 0.01 vs. MRP).

### Modulation of Gut Microbiota and SCFA Production by MRP and MRPF Treatment

**Fig. 4:**
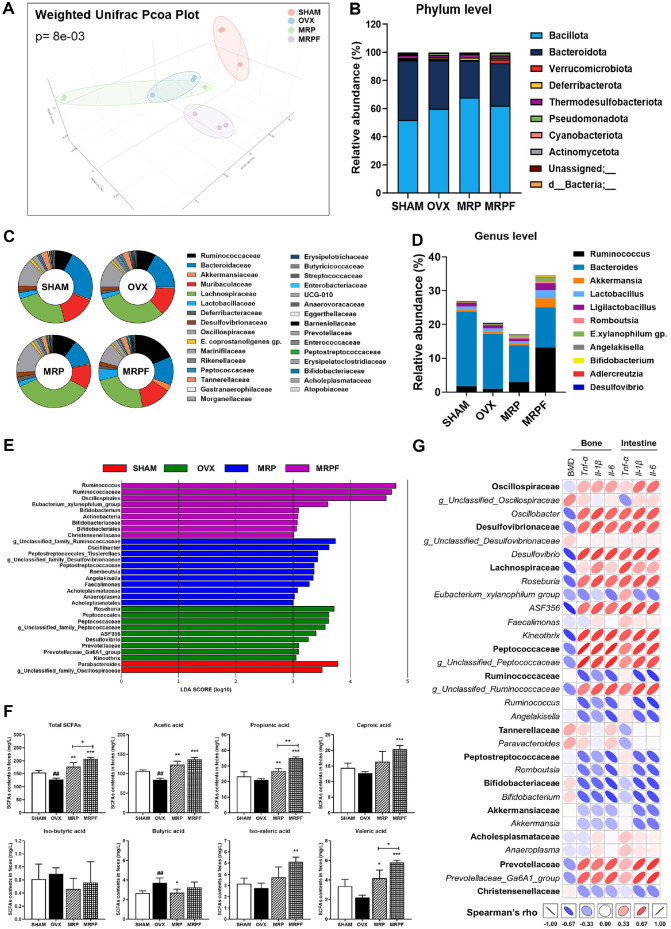
Effects of soy protein Maillard reaction product (MRP) and MRPF on gut microbiota and fecal shortchain fatty acids (SCFAs) in OVX-induced mice. (**A**) Principal coordinates analysis (PCoA) based on weighted UniFrac distance performed to evaluate beta diversity among experimental groups. (**B–D**) Microbial composition analysed at the phylum, family, and genus level. (**E**) Linear discriminant analysis effect size (LEfSe) used to identify differentially abundant taxa across groups, using an LDA score threshold of 2.0. (**F**) Fecal concentrations of SCFAs (acetic, propionic, caproic, isobutyric, butyric, iso-valeric, and valeric acids) measured using gas chromatography with flame ionization detection (GC-FID). Heptanoic acid was used as an internal standard. (**G**) Spearman’s correlation between representative gut microbial taxa and biological markers. Data are presented as mean ± standard deviation (n = 3). *p < 0.05, **p < 0.01, ***p < 0.001; ^#^*p* < 0.05, ^##^*p* < 0.01, ^###^*p* < 0.001 vs. control. Statistical differences between two groups were analyzed using Student’s *t*-test, and multiple group comparisons were performed using one-way analysis of variance. MRP, soy protein Maillard reaction product; MRPF, fermented Maillard reaction product; OVX, ovariectomized.

PCoA based on weighted UniFrac distances demonstrated distinct clustering among the groups (Fig. 4A). The OVX group was separated from the SHAM group, indicating an ovariectomy-induced shift in microbial composition. Both MRP and MRPF treatments partially shifted the microbial composition toward that of the SHAM group, with MRPF exhibiting a more pronounced restoration. PERMANOVA analysis confirmed significant differences between groups (*p* = 0.008). Taxonomic profiling at the phylum, family, and genus levels revealed notable compositional differences (Fig. 4B–4D). At the phylum level, OVX mice displayed a higher relative abundance of *Bacillota* (formerly *Firmicutes*) and a lower abundance of *Bacteroidota* than SHAM mice, indicating dysbiosis induced by estrogen deficiency. The relative abundance of *Deferribacterota* and *Verrucomicrobiota* was increased in the MRP and MRPF groups, respectively. At the family level (Fig. 4C), OVX mice exhibited elevated proportions of *Lachnospiraceae* and *Deferribacteraceae*, accompanied by a decrease in *Bacteroidaceae* and *Muribaculaceae*. In contrast, MRPF treatment increased the relative abundance of several beneficial families, including *Ruminococcaceae*, *Akkermansiaceae*, and *Lactobacillaceae*. At the genus level (Fig. 4D), MRPF administration markedly increased the relative abundances of health-associated genera such as *Ruminococcus*, *Akkermansia*, *Lactobacillus*, *Ligilactobacillus*, and *Bifidobacterium*, while reducing potentially pro-inflammatory taxa including *Desulfovibrio* and *Angelakisella*.

LEfSe analysis identified differentially abundant taxa among the experimental groups (Fig. 4E). The SHAM group was enriched in *Parabacteroides* and g__Unclassified_*Oscillospiraceae*. In contrast, the OVX group exhibited an increased abundance of inflammation-associated taxa such as *Roseburia*, *Peptococcaceae*, *Desulfovibrio*, *Prevotellaceae*, and *Kineothrix*. The MRP group showed enrichment of g_Unclassified_*Ruminococcaceae*, *Oscillibacter*, *Peptostreptococcales*-*Tissierellales*, and *Angelakisella*. The MRPF group demonstrated a greater enrichment of beneficial taxa, including *Ruminococcaceae*, *Ruminococcus*, *Oscillospirales*, *Bifidobacterium*, *Bifidobacteriaceae*, *Eubacterium_xylanophilum*_group, and *Christensenellaceae*, suggesting enhanced restoration of eubiotic composition.

Next, we assessed the fecal SCFA concentrations (Fig. 4F). The findings showed decreased levels of total SCFAs, particularly acetic acid, in OVX mice compared with those in the SHAM mice. MRPF significantly increased the concentrations of major SCFAs, including acetate and propionate.

Spearman’s correlation analysis explored the associations between gut microbial taxa and osteoimmunological parameters (Fig. 4G). *Bifidobacterium* and *Christensenellaceae* showed strong negative correlations with both bone and intestinal pro-inflammatory markers (*Tnfa*, *Il1b*, *Il6*), while exhibiting positive correlations with BMD, suggesting their potential protective role in both intestinal and bone health. Similarly, *Ruminococcus*, *Ruminococcaceae*, *Angelakisella*, *Akkermansia* and *Akkermansiaceae* were negatively associated with pro-inflammatory cytokines. Conversely, *Oscillobacter*, *Desulfovibrio*, *Lachnospiraceae*, *Roseburia*, ASF356, *Kineothrix*, g_Unclassified_*Peptococcaceae*, g_Unclassified_*Ruminococcaceae*, and *Prevotellaceae*_Ga6A1_group were positively correlated with intestinal and bone pro-inflammatory markers and negatively associated with BMD, implying their potential involvement in inflammation-mediated bone resorption under estrogen-deficient conditions.[Fig F5]

## Discussion

Menopause-associated estrogen deficiency is a principal contributor to osteoporosis in ageing women, with global prevalence rates reaching 10–40% depending on ethnicity and age [33, 34]. The imbalance between bone resorption and formation significantly elevates the risk of fragility fractures, contributing to a burden on quality of life and healthcare systems. In the present study, we investigated the bone-protective potential of soy protein-derived Maillard reaction products fermented with *L. rhamnosus* IM18 (*i.e.*, MRPF group), focusing on their ability to attenuate bone resorption in OVX mice by modulating TNF-α expression and altering the gut microbiota.

Compared with the ISP, MRP exhibited improved functional properties, which can be attributed to the formation of various Maillard reaction compounds, including reductones and small-chain sugars. Reductones, responsible for reducing power, are produced through the thermolysis of Amadori products during the intermediate stages of the Maillard reaction, along with the hydroxyl and pyrrole groups of advanced MRPs [35, 36]. Additionally, small-chain sugars such as glucose exhibit low steric hindrance, facilitating the formation of heterocyclic melanoidins that act as hydrogen donors to improve radical scavenging activity [37]. Bioactive fractions derived from glucose–lysine mixtures have demonstrated anti-inflammatory effects by suppressing transcription of nuclear factor kappa B (NF-κB) and inhibiting iNOS protein translation [19].

Fermentation of MRP by *L. rhamnosus* IM18 further enhanced its antioxidant and anti-inflammatory properties, likely due to the generation of bioactive peptides during fermentation. *L. rhamnosus* strains are known to produce low-molecular-weight peptides with strong antioxidant and anti-inflammatory properties. For instance, H. Guo *et al*. [38] reported enhanced ABTS and hydroxyl radical scavenging activity and increased oxygen radical absorbance capacity values in peptide fractions from whey protein fermented by *L. rhamnosus* B2-1. These findings align with our previous work demonstrating that fermentation of glycated casein by *L. rhamnosus* 4B15 significantly enhanced antioxidative capacity and intestinal anti-inflammatory responses [23]. Similarly, Chun *et al*. [39] reported that fermented whey–galactose conjugates inhibited the phosphorylation of extracellular signal-regulated kinase and c-Jun N-terminal kinase in LPS-induced inflammatory responses, thereby suppressing nitric oxide production and downregulating the expression of *Tnf-a* and *Ptgs2*. These findings support the potential involvement of MRPF-derived peptides in modulating inflammation-related pathways.

Furthermore, both MRP and MRPF significantly inhibited RANKL-induced osteoclastogenesis in RAW 264.7 cells, as indicated by a reduction in TRAP-positive multinucleated cells, decreased TRAP enzymatic activity, and downregulation of key osteoclastogenic markers such as *Nfatc1*. Moreover, MRPF showed greater inhibitory activity than MRP, highlighting the role of fermentation-generated bioactive components, particularly peptides in suppressing osteoclast differentiation. This finding is consistent with previous studies demonstrating osteoclast-inhibitory effects of peptides derived from diverse protein sources [40-42].

MRPF supplementation significantly improved trabecular architecture and increased BMD, Tb.N, Conn.D, and BV/TV in the OVX mouse model. These structural improvements were accompanied by reduced expression of osteoclastogenic and pro-inflammatory genes [*Tnf-a*, *Il-1b*, *Il-6*, and *Tnfsf11* (which encodes RANKL)]. Chronic inflammation is a recognized driver of osteoclastogenesis, with cytokines such as TNF-α and IL-1β directly or indirectly enhancing RANKL expression, promoting bone resorption [9, 43]. MRPF treatment also upregulated *Tnfrsf11b* (encodes OPG protein), a decoy receptor for RANKL that inhibits RANK–RANKL interaction, thereby suppressing osteoclast formation [44]. Collectively, our results demonstrate that MRPF exerts its osteoprotective effects through modulation of osteoimmune signaling, particularly by suppressing inflammatory cytokine expression and rebalancing the RANKL/OPG axis.

Estrogen deficiency is further associated with impaired gut barrier function, chronic low-grade inflammation, and gut microbiota dysbiosis, contributing to systemic inflammation and bone loss [45-49]. In this study, MRPF supplementation effectively reduced the expression of pro-inflammatory cytokines (*Tnf-a*, *Il-1b*, and *Il-6*) in femoral tissue and suppressed intestinal inflammatory markers (*Tnf-a*, *Il-1b*, *Il-6*, and *Il-17*), while upregulating anti-inflammatory mediators (*Il-10* and *Ifng*). MRPF also restored the expression of tight junction-associated genes (*Ocln*, *Cldn1*, and *Tjp1*), essential for maintaining epithelial barrier integrity, the downregulation of which is a hallmark of increased intestinal permeability [50, 51]. These findings indicate that MRPF confers both systemic anti-inflammatory and gut-protective effects under estrogen-deficient conditions, which likely contribute to its beneficial influence on bone.

On the other hand, microbiota analysis further supported our speculations. Specifically, MRP and particularly MRPF reversed OVX-induced microbial dysbiosis, as revealed by PCoA and taxonomic profiling. MRPF supplementation markedly increased the abundance of beneficial genera, including *Ruminococcus*, *Akkermansia*, *Lactobacillus*, and *Bifidobacterium*, which negatively correlated with inflammatory cytokines, such as TNF-α. *Ruminococcus* was previously shown to be negatively associated with NF-κB /MAPK signaling-related markers, including *Tnf-a*, *Il-1b*, *Il-6*, and positively linked to *Ocln*, *Cldn1*, and *Tjp1* [52]. *Akkermansia* enhances gut barrier function and reduces TNF-α production in inflammation-related diseases [53, 54]. *Lactobacillus* and *Bifidobacterium* are widely recognized probiotics known to modulate immune responses and suppress pro-inflammatory cytokines [55-58]. Many of these taxa have been independently linked to bone turnover and gut permeability. For example, *Akkermansia* and *Lactobacillus* enhance barrier integrity and suppress osteoclastogenic T-cell signaling, whereas *Bifidobacterium* reduces TNF-α/NF-κB–mediated bone resorption; conversely, *Desulfovibrio* and *Prevotellaceae* exacerbate gut leakiness and promote osteoclast activation through inflammatory cytokine pathways [59-62]. MRPF also mitigated OVX-induced increases in taxa such as *Roseburia*, *Desulfovibrio*, *Prevotellaceae*, and *Kineothrix*, associated with estrogen deficiency, bone resorption, and mucosal inflammation. For instance, *Roseburia* abundance correlates with increased TRAP activity and osteoclastogenesis [63], while *Desulfovibrio*, a predominant sulphate-reducing bacterium, is elevated in inflammatory bowel disease and correlates with inflammatory cytokines such as TNF-α, IL-1B, and IL-17A, and bone resorption markers such as CTX-1, suggesting its contribution to leaky gut and systemic inflammation [58, 64]. *Prevotellaceae* abundance was negatively correlated with oestradiol levels in menopausal women [65], and *Kineothrix*, producing saccharolytic butyrate, displayed significantly lower relative abundances in estrogen-supplemented OVX rats than that in estrogen-deficient controls, suggesting estrogen responsiveness despite its butyrate-producing and saccharolytic characteristics [66, 67]. These findings collectively align with the LEfSe and Spearman’s correlation analysis, reinforcing the notion that MRPF supplementation exerts a more pronounced microbiota-modulating effect in estrogen-deficient mice, restoring gut dysbiosis and suppressing inflammation-associated microbes. Furthermore, MRPF increased SCFA levels, particularly acetate and propionate, which enhance absorption of Ca^2+^ and Mg^2+^, promote regulatory T cells (also known as Tregs) differentiation, suppress pro-inflammatory CD4^+^ effector T cells, and regulate bone remodeling by stimulating osteoblasts and inhibiting osteoclast precursors [68]. These findings indicate that SCFAs may contribute to improved bone and immune function.

Taken together, these findings indicate that the enhanced efficacy of MRPF over MRP is attributable to molecular changes introduced during fermentation, most notably the formation of low-molecular-weight bioactive peptides. These peptides possess well-documented antioxidant and anti-inflammatory activities that mitigate oxidative stress and cytokine-driven signaling associated with accelerated osteoclastogenesis [69]. Accordingly, the stronger radical-scavenging capacity and more pronounced suppression of inflammatory mediators observed in the MRPF group offer a plausible explanation for its greater osteoprotective effects. Fermentation-derived peptides have also been reported to enhance epithelial barrier integrity and restore gut microbial composition [70], which is consistent with the improved tight-junction gene expression and the microbiota restoration observed in MRPF-treated OVX mice. This peptide-centered mechanism provides a coherent biological basis for the multifaceted improvements induced by MRPF and aligns with the peptide profile identified in our subsequent analysis.

Our peptide profiling identified 28 peptides generated during fermentation, including several with known antioxidant or ACE-inhibitory functions [71-74]. Particularly, TWNPNNKPF (P6; the most abundant peptide), DSHQKIRHF (P10), and NALKPDNRIE (P11) have known antioxidant activities, while SFLVPPQESQ (P7) and PSFLVPPQESQRR (P27) exhibit angiotensin-converting enzyme inhibitory effects [38, 75-77]. These peptides may exert local effects in the gut by modulating inflammation and microbial composition, ultimately contributing to the observed systemic bone-protective outcomes. Further validation works are warranted for elucidating the roles of the peptides.

There are several limitations to be noted. First, although OVX mice are a widely accepted model for postmenopausal osteoporosis, the findings may not fully translate to human physiology due to the nature of the experimental approach. Second, while MRPF-derived peptides were identified, their individual bioactivities were not directly confirmed *in vivo*. Importantly, however the decision to evaluate the MRPF peptide mixture as a whole—rather than testing each peptide in isolation—reflects the physiological reality where these peptides may act synergistically, and thus a holistic assessment may more accurately capture the in vivo protective effect observed. Third, the causal relationships between specific microbial taxa and bone-protective effects require further elucidation. Future studies addressing these limitations will enhance the understanding of therapeutic potential of MRPF.

## Conclusion

This study demonstrates that dietary supplementation with MRPF derived from soy protein exerts potent anti-inflammatory and bone-protective effects in an estrogen-deficient mouse model. Specifically, the MRPF supplementation suppressed key pro-inflammatory cytokines such as TNF-α, in both intestinal and bone tissues, restored gut barrier integrity, and modulated gut microbiota composition. These effects were closely associated with the generation of bioactive peptides during *L. rhamnosus* IM18-mediated fermentation, several of which are known for their antioxidant and ACE-inhibitory activities. Collectively, these findings reveal that MRPF mitigates gut-derived systemic inflammation and contributes to the prevention of bone resorption. Together, these findings indicate that MRPF is a promising functional food ingredient for managing postmenopausal osteoporosis through modulation of the gut–bone axis via the suppression of inflammatory markers, such as TNF-α, and the remodeling of gut microbiota.

## Supplemental Materials

Supplementary data for this paper are available on-line only at http://jmb.or.kr.



## Figures and Tables

**Fig. 5 F5:**
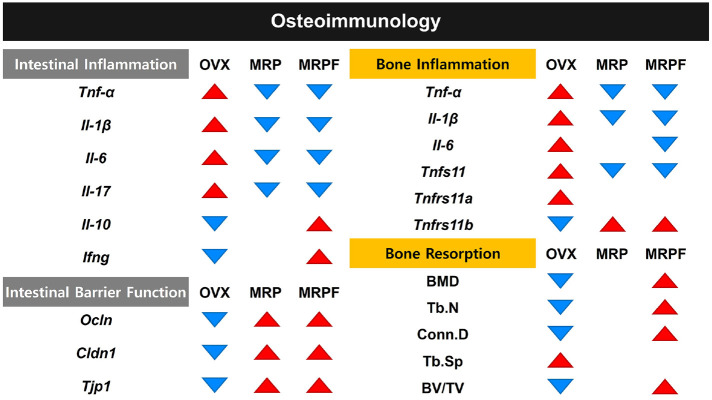
Schematic summary of the effects of MRP and MRPF on osteoimmunological parameters. Red upward triangles (▲) indicate an increase, while blue downward triangles (▼) indicate a decrease in gene expression or biological function. The diagram illustrates OVX-induced changes and the modulatory effects of MRP and MRPF on intestinal inflammation, intestinal barrier function, bone inflammation, and bone resorption. MRP, soy protein Maillard reaction product; MRPF, fermented Maillard reaction product.

**Table 1:** Peptide list of MRPFa.