# Supplementary material for: Fermented Soy Protein Maillard Product Prevents Bone Loss via TNF-α Suppression and Gut–Bone Axis Modulation in Ovariectomized Mice
Source: J Microbiol Biotechnol. 2025 Dec 29;35:e2511004. doi: 10.4014/jmb.2511.11004 (PMC12790997; doi:10.4014/jmb.2511.11004)
Supplement: Supplementary file 1 [file jmb-35-e2511004-supple.pdf]

J. Microbiol. Biotechnol. 2025. 35: e2511004

https://doi.org/10.4014/jmb.2511.11004

Supplementary Table and Figure

Fermented soy protein Maillard product prevents bone loss via TNF-α suppression and gut–bone axis modulation in ovariectomized mice

Hyun Jin Bae[†,1], Jae Yeon Joung[†,2,3], Hyo Su Choi [†,1], Ji Seung Han[1], Jin Hwan Kim[1], Jae Kyeom Kim[1,4], and Nam Su Oh[*,1]

[1] Department of Food and Biotechnology, Korea University, Sejong 30019, Republic of Korea

[2] College of Life Sciences and Biotechnology, Korea University, Seoul 02841, Republic of Korea

[3] Institute of Life Sciences and Natural Resources, Korea University, Seoul 02841, Republic of Korea

[4] Department of Health Behavior and Nutrition Sciences, University of Delaware, Newark, DE 19701, USA

[†] Hyun Jin Bae, Jae Yeon Joung, and Hyo Su Choi contributed equally to this study.

[*] **Corresponding author: Nam Su Oh, PhD**

E-mail: klanvin@korea.ac.kr

Table S1. Primer sequences used in this study

Gene	Forward Primer (5' to 3')	Reverse Primer (5' to 3')
Tnf-α	CTGAACTTCGGGGTGATCGG	GGCTTGTCACTCGAATTTTGAGA
Il-1β	CAACCAACAAGTGATATTCTCCATG	GATCCACACTCTCCAGCTGCA
Il-6	AAGTCGGAGGCTTAATTACACATGT	CCATTGCACAACTCTTTTCTCATTC
Nos2	CCCTTCCGAAGTTTCTGGCAGCAGC	GGCTGTCAGAGAGCCTCGTGGCTTTGG
Ptgs2	TGTATCCCCCCACAGTCAAAGACAC	GTGCTCCCGAAGCCAGATGG
Nfatc1	CCCGTCACATTCTGGTCCAT	CAAGTAACCGTGTAGCTGCACAA
Acp5	ACTTCCCCAGCCCTTACTACCG	TCAGCACATAGCCCACACCG
Esr1	CCTCCCGCCTTCTACAGGT	CACACGGCACAGTAGCGAG
Esr2	CTCTTCCCAGCAGCAGTCAGTC	AGCATCTCCAGCAGCAGGT
Tnfs11	CAGCATCGCTCTGTTCCTGTA	CTGCGTTTTCATGGAGTCTCA
Tnfrsf11a	CCAGGAGAGGCATTATGAGCA	ACTGTCGGAGGTAGGAGTGC
Tnfrs11b	GTTTCCCGAAGGACCACAAT	CCATTCAATGATGTCCAGGAG
Il-17	CCACCTCACACGAGGCACAAGT	TCAGCAGCAGCAACAGCATCAGA
Il-10	CTTACTGACTGGCATGAGGATCA	GCAGCTCTAGGAGCATGTGG
Ifng	TGAACGCTACACACTGCATCTTGG	CGACTCCTTTTCCGCTTCCTGAG
Ocln	TCGCTTATCTTGGGAGCCTG	TTCAAAAGGCCTCACGGACA
Cldn1	CCCCATCAATGCCAGGTATG	TTGTTTTCCGGGGACAGGAG
Tjp1	GTTGGTACGGTGCCCTGAAAGA	GCTGACAGGTAGGACAGACGAT
Gapdh	CATCACTGCCACCCAGAAGACTG	ATGCCAGTGAGCTTCCCGTTCAG

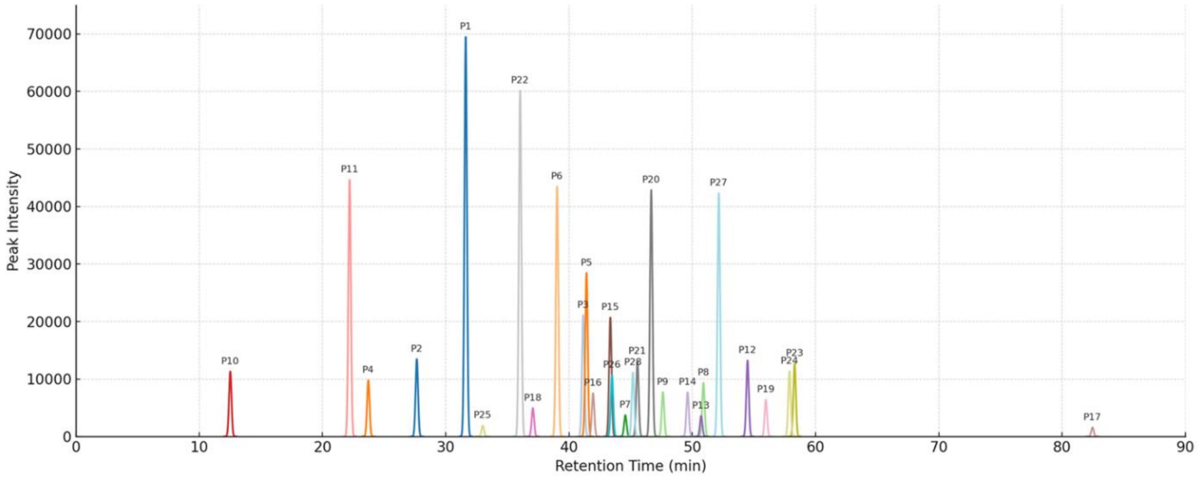

Fig. S1. Overlayed XIC peaks of 28 peptides derived from fermented ISP with *Lacticaseibacillus rhamnosus* IM18. Each peak represents an individual peptide identified via LC-MS/MS analysis, plotted by retention time (min) and MS signal intensity. Peaks are labeled with peptide numbers corresponding to Table 1.
